# Iron, Magnesium, Vitamin D, and Zinc Deficiencies in Children Presenting with Symptoms of Attention-Deficit/Hyperactivity Disorder

**DOI:** 10.3390/children1030261

**Published:** 2014-09-29

**Authors:** Amelia Villagomez, Ujjwal Ramtekkar

**Affiliations:** 1 University of Arizona, 2800 E. Ajo Way Suite 300, Tucson, AZ 85713, USA; 2 Mercy Children’s Hospital, 621 S. New Ballas Road, Suite 693A, Saint Louis, MO 63141, USA; E-Mail: ujjwal.ramtekkar@mercy.net

**Keywords:** attention-deficit/hyperactivity disorder, zinc, magnesium, vitamin D, ferritin, iron

## Abstract

Attention-Deficit/Hyperactivity Disorder (ADHD) is a neurodevelopmental disorder increasing in prevalence. Although there is limited evidence to support treating ADHD with mineral/vitamin supplements, research does exist showing that patients with ADHD may have reduced levels of vitamin D, zinc, ferritin, and magnesium. These nutrients have important roles in neurologic function, including involvement in neurotransmitter synthesis. The aim of this paper is to discuss the role of each of these nutrients in the brain, the possible altered levels of these nutrients in patients with ADHD, possible reasons for a differential level in children with ADHD, and safety and effect of supplementation. With this knowledge, clinicians may choose in certain patients at high risk of deficiency, to screen for possible deficiencies of magnesium, vitamin D, zinc, and iron by checking RBC-magnesium, 25-OH vitamin D, serum/plasma zinc, and ferritin. Although children with ADHD may be more likely to have lower levels of vitamin D, zinc, magnesium, and iron, it cannot be stated that these lower levels *caused* ADHD. However, supplementing areas of deficiency may be a safe and justified intervention.

## 1. Introduction

Attention-deficit/hyperactivity disorder (ADHD) is a neurodevelopmental disorder characterized by impaired levels of inattention and hyperactivity in more than one setting. The rates of ADHD have risen over the past few decades and continue to rise. According to recent data from the National Survey of Children’s Health, 11% of US children ages 4–17 had been diagnosed at some point in their lives with ADHD; this is a 42% increase from the 7.8% of children in 2003. By parent-report, the percentage of children aged 4–17 taking medication for ADHD was 4.8% in 2007 and 6.1% in 2011 [1].

There is no single cause of ADHD, and much research has focused on both environmental and genetic risk factors independently. Recent studies also demonstrate the complex interplay of genetic and environmental factors. For example, a genetically endowed variability of dopaminergic genotypes to specific environmental risk factors, such as prenatal smoking, can result in a specific subtype of ADHD [2]. ADHD has been associated with maternal smoking, alcohol and substance misuse, maternal stress, low birth weight and prematurity, organophosphates, polychlorinated biphenyls, lead, artificial food colorings, severe early deprivation, and family adversity. Although these factors have been correlated with ADHD and some are risk factors, none of them have been shown to definitively cause ADHD [3]. Although medications for ADHD have a large effect size and psychosocial interventions can augment treatment success, more than 30% of children are still symptomatic despite combined treatment [4].

Many parents choose not to start medication for fear of side effects and may seek “alternative” or “natural” treatments. Data from the National Health Interview Study demonstrated that for children ages 7 to 17, by parent-report, 8.9% of children had been diagnosed with ADHD, and of those, 24.7% used at least one type of complementary and alternative medical (CAM) therapy [5]. There is no conclusive data to support nutrient deficiencies as a cause of ADHD. However, research does exist demonstrating that patients with ADHD have reduced levels of vitamin D, zinc, ferritin, and magnesium. We will review the existing literature on zinc, ferritin, magnesium, and vitamin D and their association with ADHD to guide clinicians on possible appropriate laboratory screening measures for children presenting with symptoms consistent with ADHD.

## 2. Zinc

### 2.1. Role of Zinc

Zinc plays an important role for protein and DNA synthesis, in wound healing, for bone structure, and on the immune system. Deficiency of zinc can result in poor growth and retarded development, hair loss, diarrhea, suppression of aspects of cell-mediated immunity, and dermatitis. There is no pathognomonic sign for zinc deficiency and severe zinc deficiency is uncommon in the United States [6,7].

Zinc is considered a trace element because plasma concentration is only 12–16 μM; the body contains approximately 2–4 grams of zinc, the majority of which is in the bone and skeletal muscle. Only 0.1% of the total amount of zinc in the body is in the plasma [8]. The body does not store zinc, so supply must be repleted by dietary intake [9]. 

The Dietary Reference Intake (DRI) is the general term for a set of reference values used to plan and assess nutrient intakes of healthy individuals. The current DRI is based on the previous Recommended Daily Allowance (RDA) and is 5 mg for females and males 4–8 years old, 8 mg for females and males 9–13 years old, 9 mg for females 14–18 years old, and 11 mg for males 14–18 years old. The RDA is the amount of intake adequate for 97%–98% of the population; therefore, for 2%–3% of the population, this intake may not be sufficient [10]. In the US, meat accounts for 50% of dietary zinc intake, legumes and cereals account for 30%, and dairy products about 20% [11]. 

Although plant sources contain zinc, they are also high in phytic acid, which binds to zinc, forms an insoluble compound and decreases its bioavailability. According to the Institute of Medicine’s report, vegetarians may require as much as a 50% greater intake of zinc given that the major source in the diet is grains and legumes which contain a high amount of phytic acid [8]. Inorganic iron and calcium supplements may decrease zinc absorption as well as alcohol, infection and surgery. Animal studies show that excessive dietary intake of calcium decreases zinc absorption, however, studies have not been done in humans.

The Tolerable Upper Intake (UL), the maximum daily intake for the general population that is unlikely to cause adverse side effects is: 12 mg for 4–7 years of age, 23 mg for 9–13 years of age, and 34 mg for 14–18 years of age. [8] There is a small window between the DRI and the UL of zinc. In adults, acute adverse side effects of excessive zinc may occur at 50–150 mg/day and include epigastric pain, nausea, loss of appetite, abdominal cramps, diarrhea and headache [8]. Chronic adverse side effects associated with excessive zinc supplementation include immune suppression, and decreased HDL cholesterol [8,12,13].

In the Health Professionals Follow-Up Study, supplemental zinc intake below 100 mg/day was not associated with increased prostate cancer risk, however men taking more than 100 mg/day had a 2.29 relative risk of advanced prostate cancer [14]. Zinc and copper compete for absorption; therefore there is a concern for copper deficiency during zinc supplementation. However, the threshold for this effect is unknown. Maret and colleagues offered the practical suggestion that until further research on this topic is done, the ratio of zinc to copper should not exceed 10–12 [7]. Sequelae of copper deficiency can include: reduced skin pigmentation, central nervous system impairment, osteoporosis, decreased immune function, anemia, and fainting spells [6].

### 2.2. Relevance for ADHD

Although the exact mechanism of how zinc may contribute to symptoms of ADHD is not known, it is a cofactor for more than 300 enzymes and is involved in the pathway for the body’s production of prostaglandins and neurotransmitters [9]. Zinc is necessary for B6 to be metabolized to its active form, pyridoxal phosphate, which in turn plays a role in conversion of tryptophan to serotonin [15]. Zinc assists in both the production and regulation of melatonin; melatonin is an important factor in the pathophysiology of ADHD due to its modulation of dopamine [16,17,18]. Zinc also binds to and regulates the dopamine transporter, which is a site of action of psychostimulants used to treat ADHD [18]. However, there is no clear evidence that zinc deficiency directly results in alteration of the melatonin or dopamine transporter in children with ADHD.

There is a bidirectional association between zinc and essential fatty acids (EFAs). Zinc serves as a coenzyme of delta-6-desaturase, an enzyme crucial for EFA metabolism [19,20]. Prostaglandin E2, a product of essential fatty acid metabolism, facilitates the absorption of zinc in the gut [21]. Individuals with ADHD have been shown to have altered red blood cell fatty acid profiles, and supplementation with EFAs have been shown to have a small but significant effect for the treatment of ADHD [22,23,24].

### 2.3. Reduced Levels in ADHD

In 1981, Colquhoun and Bunday measured hair samples of 46 hyperactive children and noted that 31 had levels below the normal range; they hypothesized that zinc deficiency results in decreased levels of essential fatty acids and may contribute to hyperactivity [25]. In a Turkish study, there was a statistically lower serum zinc level in the ADHD group compared to healthy controls: 60.6 ± 9.9 µg/dL *vs.* 105.8 ± 13.2 µg/dL, respectively. Free fatty acid (FFA) levels in the ADHD group were one third of those in the control group (ADHD group: 0.176 ± 0.102 mEq/L and in control group: 0.562 ± 0.225 mEq/L with *p* < 0.001). There was a correlation between serum FFA and zinc levels. Authors postulated that the FFAs were possibly low secondary to zinc deficiency [26].

In a small study of 18 ADHD boys (ages 6–12) entering a double-blind balanced crossover comparison between one month of dextro-amphetamine and one month of placebo, there was no statistically significant difference between baseline hair zinc in ADHD *vs.* normal controls. However, higher baseline hair zinc levels predicted a greater placebo-controlled amphetamine improvement. The authors hypothesized that those with higher zinc levels had a sufficient amount of zinc for the stimulants to be effective and were not symptomatic because of zinc deficiency but rather from other causes. They further postulated that those with low levels of zinc would require zinc supplementation for an optimal stimulant response [27].

Researchers in British Columbia measured non-fasting serum zinc levels and evaluated 3-day food records on 43 children aged 6–12 with ADHD (18 taking stimulants, 9 atomoxetine, and 17 drug naïve). In this affluent sample (all but two came from homes with household income greater than $60,000), 28% and 61% aged 6–8 and 9–12 respectively did not meet the Estimated Average Requirement (EAR) for zinc. EAR is the average daily nutrient intake estimated to meet the requirements of half the healthy individuals in a particular age and gender group. The prevalence of zinc deficiency in children with ADHD in this study population was 8 times greater than the reported prevalence of zinc deficiency for normal populations (3.3% for males and 3% for females) [28]. Twenty-six percent of the children aged 6–8 and 20% of the 9–12 year olds had serum zinc levels below the 2.5th percentile of the National Health and Nutrition Examination Survey (NHANES II) cutoffs for zinc deficiency. NHANES II is a survey research program conducted by the National Center of Health Statistics (NCHS) to assess the health and nutritional status of adults and children in the United States and also forms the basis for national standards for such measurements.

A research study done in the US showed that in children with ADHD, lower serum zinc levels were associated with greater parent and teacher ratings of inattention (*r* = −0.45). However, this same correlation was not present when examining symptoms of hyperactivity/impulsivity of ADHD—there was actually a correlation in the positive direction, although insignificant and small (*r* = 0.14; *p* = 0.35) [29].

A Turkish study measured the event-related potentials (ERPs) for 3 groups: those with low zinc (N = 13, zinc level < 80 μg/dL) and ADHD, those with normal zinc levels (N = 14, zinc level ≥ 80 μg/dL) and ADHD, and age-matched controls without ADHD (N = 24). The plasma zinc levels were significantly lower in both ADHD groups as compared to the group without ADHD (means were 65.8 μg/dL in low zinc group and 89.5 μg/dL in zinc non-deficient group *vs.* control mean: 107.8 μg/dL). On EEG, compared to the zinc non-deficient ADHD group, the low zinc ADHD group had significantly shorter N2 latency in the frontal and parietal regions. Additionally, data demonstrated a significant positive correlation between plasma zinc levels of children with ADHD and the amplitude and latencies of N2 in the frontal region. In the context that prior work has suggested that N2 wave changes may reflect an atypical inhibition process, authors suggested that zinc deficiency may have a deleterious effect on inhibitory control in patients with ADHD [30,31].

Although not all studies have shown lower levels of zinc in children with ADHD, a systemic review looking for biomarkers for ADHD concluded that studies have found lower levels of zinc when measured in serum, plasma, urine, and hair [32]. The meta-analysis showed significantly lower levels of zinc among patients with ADHD (*d* = −0.88), however it was limited because of significant heterogeneity (*p* = 0.0002, *I*^2^ = 79%) [33].

### 2.4. Etiology of Lower Levels of Zinc

Zinc levels may be lower in patients with ADHD secondary to differences in dietary intake, differences in absorption, or other mechanisms [28,34]. Low zinc levels have also been associated with depression in adults [1,35]. Raison hypothesized that both Major Depressive Disorder and low zinc levels may have a common denominator: inflammation. Perhaps the drop of zinc levels is an evolutionarily acquired mechanism the body uses when trying to fight infections: less zinc means less nutrients for pathogens to utilize [3,36]. Although inconsistent, there is evidence that pro-inflammatory markers are elevated in children with ADHD [4,37]. Stress, acute trauma, and infection cause changes in hormones (e.g., cortisol) and cytokines (e.g., interleukin 6) that cause sequestration of zinc in the liver and spleen and consequently lower plasma zinc concentration [5,38,39]. Therefore, it is plausible that lower levels of zinc were seen in patients with ADHD because of an increased pro-inflammatory state.

An alternate explanation could involve increased zinc-wasting in the urine of children with ADHD. In a study by Ward and colleagues, children who were classified as “hyperactive” were given a drink with one of either 3 different types of artificial food coloring; their response was compared to children without hyperactivity. Hyperactive children ingesting the drink with sunset yellow (*n* = 12) or the tartrazine (*n* = 23) had significantly decreased levels of plasma zinc and an increase in urinary zinc as compared to controls. This led investigators to postulate that hyperactive children may be more sensitive to food coloring possibly secondary to causing zinc-wasting in urine for individuals with hyperactivity [6,7,40].

### 2.5. Interpreting Zinc Measurements

Only 0.1% of total body zinc is in the plasma and its concentration is tightly regulated by homeostatic mechanisms. Plasma levels may not reflect intracellular and zinc status in tissues throughout the body [8,9], and may show changes only when depletion is severe or extended [7,9,38].

A systematic review of the literature concluded that plasma zinc concentrations did respond to supplementation; however, no definitive conclusions could be made regarding its ability to change in response to marginal dietary intake [10,41]. Serum and plasma levels are virtually equivalent [11,42]. Although serum/plasma zinc are usually used as markers of zinc status in populations studies, and are accessible and convenient, there is currently no single universally accepted method to measure total body zinc status [8,43].

Frank deficiency of zinc may be detected by serum/plasma levels, but for marginal deficiency, levels may be imprecise and insensitive. Therefore, physical signs and functional affects may occur in the absence of low levels.

Additionally, independent of zinc status, plasma levels can be affected by infection, stress, low serum albumin, oral contraceptive use, steroid use, diarrhea, and phase of the menstrual cycle [8,38]. Additionally, zinc levels in the serum/plasma vary throughout the day. In a population of adult women, hour plasma zinc samples were taken, and patients were provided with standard meals at standard times. Levels decreased 30–90 min after a meal, reached the lowest point 3–4 h after the meal, increased throughout the night, and peaked in the morning before breakfast. Highest values of the day (morning) were 22% higher than lowest value at 2100 [8,44]. Various studies have used different cut offs for determining zinc deficiency. Based on NHANES data, a suggested lower cut off of serum zinc concentration of between 56 and 74 μg/dL was given (depending on fasting status and age) [8,12,13,38].

Given the limitations of measuring plasma/serum zinc alone, some researchers also measured hair and urine zinc to better ascertain zinc status in the body [14,15]. Arnold re-analyzed data from a previous double-blind, placebo-controlled crossover comparison of d–amphetamine and Efamol (evening primrose, containing 320 mg of gamma-linolenic acid per day) for treatment of ADHD to determine if there was a differential response moderated by zinc status. Gamma-linolenic acid is an omega-6 fatty acid and a precursor of the series 2 prostaglandins, which facilitates zinc absorption [7,21]. Status of zinc in the body was classified as adequate, borderline, or frank deficiency by considering levels of hair, urine, and red blood cell zinc. Hair levels were considered an indicator of long-term zinc status. Urine and red cell zinc were considered indicators of recent zinc intake. High levels of urine or hair zinc were considered either as a result of high dietary intake or excess wasting of normal intake. Efamol had greatest effect in the group that had borderline zinc status. Authors speculated that gamma-linolenic acid may have increased absorption of zinc in these patients with borderline deficiency [6,15].

### 2.6. Zinc as Treatment

Four studies have shown positive results for zinc in the treatment of ADHD. In Turkey, 400 boys with a mean age of 9.6 were randomized in a double blind controlled trial to zinc sulfate 150 mg (approximately 40 mg of elemental zinc) or placebo. Approximately half of subjects in both the placebo and zinc group dropped out of the trial secondary to lack of efficacy and adverse side effects. Adverse side effects were similar in the zinc and placebo group except there was a much higher incidence of children in the zinc group reporting a metallic taste in the mouth. At week 12, serum zinc levels increased as did the level of FFAs. Zinc levels increased from (88.8 ± 25.5 μg/dL) to (140.6 ± 33.6 μg/dL, *p* = 0.01) and FFA levels increased from 0.69 ± 0.39 mEq/L to 0.85 ± 0.38 mEq/L (*p* = 0.03). There was a significant improvement in hyperactivity, impulsivity, and socialization in ADHD patients; however, there was no improvement of attention. Improvement in hyperactivity, impulsivity, and social inappropriateness was more pronounced in those patients who were older, had higher BMIs, and lower pretreatment zinc and FFA values. Full therapeutic response was seen in 28.7% of the subjects in the zinc group *vs.* 20.4% in placebo group. Although the results were statistically significant, an intention-to-treat analysis was not done and dosages were in significant excess of the DRI. Authors noted that more than 70% still had significant symptoms and would likely benefit with supplementary medication [9,45].

This study was followed by a second study in Turkey, where it had been previously estimated that 20% of the children are deficient in zinc. In a double blind-randomized control trial, 226 third graders from a low socioeconomic level were studied and supplemented with 15 mg of elemental zinc syrup for 10 weeks, or a placebo. Zinc levels were drawn in a subgroup and no children were found to be deficient, however authors noted that this may have been due to an error in lab measurement. Parent rating scale showed improvement for attention deficit and hyperactivity in the zinc group but oppositional behavior deceased in the placebo group. No statistical difference in Conner’s Rating Scale for Teachers was noted. When a subgroup analysis was done, in the group whose mother’s education consisted of primary school or less, compared to placebo, there was a statistically significant decrease in the prevalence of children with clinically impairing levels of hyperactivity, attention deficit and oppositional behavior in study group. However, no differences were seen when examining the Conner’s Rating Scale for Teachers [15,46].

In Iran, 44 outpatient children ages 5–11 with combined type ADHD and who were medication naïve, were given methylphenidate plus placebo or methylphenidate plus zinc sulfate 55 mg (elemental zinc approximately 15 mg), in a double-blind trial for 6 weeks. As assessed by the Teacher and Parent ADHD Rating Scale, symptoms improved in both groups with a significant better outcome for those in the zinc group. The treatment was tolerable with only nausea being a side effect more prevalent in the zinc group, and no one dropped out of the study b/c of side effects. However, there was a lack of a full placebo group as 13 out of 22 in the zinc group complained of having a metallic taste compared to none in the control group. Zinc levels were not measured in this study [17,18,47].

Arnold and colleagues conducted a study to determine if results could be replicated in the United States. Fifty-two children, ages 5–14, participated in a three phase trial. In phase 1, children received zinc glycinate 15 mg or placebo for 8 weeks. Phase 2 consisted of two weeks of adding open-label fixed dose d-amphetamine (AMPH) to both the double-blind zinc and placebo. Phase 3 consisted of 3 weeks of AMPH titrated to optimal clinical effect. This was followed by an open extension of 8 weeks in which participants in the placebo group were started on zinc (to allow all participants to try zinc). Although there were no significant differences in teacher or parent rating scores between the zinc and placebo group in phase 1 or 2 of the trial, data from phase three showed that the group receiving zinc 15 mg twice a day required a 37% lower dosage of AMPH when compared to placebo [18,48]. Authors speculated that this trial was unable to replicate previous response rates because prior studies were done in areas of high zinc deficiency. Zinc deficiency is more common in the Middle East (as compared to the US) due to high consumption of unleavened whole-grain bread and beans. Alternatively, differences could be attributed to the use of supplemental zinc glycinate in the US trial rather than zinc sulfate used in the Mideast trials. Given concern for possible nutrient-nutrient interaction with zinc supplementation, a safety assay was done to look at ferritin, red cell hemoglobin, and ceruloplasmin; all showed no significant changes. Dietary intake (Kids’ Food Questionnaire) showed that zinc intake was at borderline minimal intake [19,20,48].

Not all studies involving zinc supplementation have produced positive results for symptoms of ADHD. In a placebo controlled trial in Guatemala, 674 children grades 1–4 in Guatemala, at risk of zinc deficiency, were randomized to zinc oxide 10 mg per day, 5 days a week *vs.* placebo for 6 months. There were no differences in mental health outcomes but serum zinc levels did increase during the study and increases were associated with decreases in internalizing symptoms but not hyperactivity or externalizing symptoms [21,49]. In a double-blind trial in Chile, 40 children with ADHD were randomized to methylphenidate + placebo *vs.* methylphenidate + zinc sulfate 10 mg/day for 6 weeks. There was no improvement in parent rating scores and a non-significant improvement in teacher’s scores of the Conners Global Index. The mean zinc levels were normal at baseline, and decreased with treatment, with less decrease in zinc group, although not statistically significant: (placebo: 95.9 ± 21.5 to 77.9 ± 15.5; zinc: 90.3 ± 9.1 to 85.0 ± 12.0 μg/dL; NS) [22,23,24,50].

### 2.7. Other Considerations

One well-known side effect of psychostimulants is appetite suppression. A group of 100 children (average age of 11.2 years) with ADHD and treated with methylphenidate-ER (METH-ER) in Spain were given a food intake survey where they reported the foods they had consumed over the previous 3 days. On average, patients had been treated with METH-ER for approximately 28 months with a mean dosage of 1.02 mg/kg/day. The children with ADHD consumed an average of 1,778 calories per day and the control group consumed 2,072 calories (*p* < 0.0001). There was a statistically significant difference in zinc intake between the medicated ADHD group and the control group: 8.6 mg (SD: 2.6) of zinc *vs.* 10.2 mg/day (SD: 2.7) [25,51]. The DRI for this age group is 8 mg; therefore, a subgroup in the ADHD group was at an increased risk of zinc-deficiency secondary to poor dietary intake.

### 2.8. Conclusion

Zinc plays an important role in neurologic functioning. ADHD has been associated with lower levels of hair, plasma, serum, and urinary zinc. Although the efficacy of supplementation with zinc is not definitively clear, there is evidence to support that many children even in developed countries have sub-optimal levels of zinc intake. Children taking stimulants and experiencing appetite suppression may have decreased caloric intake and be at an increased risk of insufficient dietary intake of zinc. There are mixed results whether baseline zinc status predicts improvement of ADHD-related symptoms with supplementation [26,45,48]. Given that plasma zinc levels are an imprecise measurement of zinc status, normal zinc levels do not indicate adequate zinc availability to the central nervous system; however, low levels indicate deficiency in the body. Measuring more than one indicator of zinc status may provide more information regarding zinc status [15,27]. Checking plasma/serum levels of zinc (suggestive of short term zinc status) and/or hair zinc (suggestive of long term zinc status) may be appropriate for individuals not responding to conventional treatments for ADHD and populations at higher risk of zinc deficiency (e.g., vegetarians) [27,28]. It has been recommended that zinc in RDA/RDI dosages as part of a balanced vitamin/mineral supplement is a safe and cost effective intervention [29,52].

## 3. Magnesium

Magnesium is involved in at least 300 enzymatic reactions, and required for fatty acid synthesis. It also plays a role in muscle relaxation, protein synthesis, and energy production. Muscles contain 27% of all the magnesium in the body and bones contain 60%. Dietary sources include: whole grains, nuts, legumes, seafood, and green vegetables [6,30,31]. In animal studies, magnesium has been shown to activate tyrosine hydroxylase, the rate-limiting step in dopamine synthesis. Magnesium also binds serotonin and dopamine to their receptors [32,53].

### 3.1. Evidence for Differential Level

According to a study done in Poland, children with ADHD had lower levels of magnesium compared to healthy controls. Percentage of deficiency was dependent on method of measurement: 33.6% were deficient on serum magnesium, 77.6% on hair, and 58.6% on red blood cell magnesium [33,54]. In an Egyptian sample, compared to healthy controls, serum magnesium levels were lower in children with hyperactive and combined type ADHD (2.2 ± 0.9 mEq/L *vs.* 1.7 ± 0.8 mEq/L, *p* = 0.02); there were no differences in levels in the subgroup of children with ADHD-inattentive type. In a sample of 76 children in France, compared to healthy controls, those with ADHD had lower RBC magnesium levels. However there was not a statistical difference of serum magnesium levels between the control and ADHD group [28,34,55]. Conversely, a group of college students diagnosed with ADHD in the United States, when compared to healthy controls, had *higher* levels of serum magnesium (789.35 ± 127.22 uM *vs.* 630.67 ± 87.28 uM, *p* = 0.002) but when RBC magnesium levels were compared, they were no differences between groups [24]. Possible hypotheses to account for conflicting results include different dietary intake between populations and methods of magnesium assessment. Blood plasma/serum magnesium do not precisely reflect intracellular level of magnesium; this may be better reflected in RBC-mag levels [56].

### 3.2. Measuring Magnesium

Only 1% of magnesium is located in the extracellular space. Therefore, plasma/serum levels may not reflect intracellular levels. Furthermore, plasma/serum levels are tightly regulated; one-third of magnesium in bone is freely exchanged with the plasma; therefore, even if intake is inadequate, normal serum levels may be maintained by bone stores. A systematic review concluded that serum/plasma magnesium concentration, red blood cell concentrations (RBC-mag), and urinary magnesium are useful markers of magnesium status in healthy populations; these markers responded to changes depletion/supplementation. It is unknown whether serum/plasma magnesium or RBC-magnesium reflects intracerebral magnesium levels [56]. In clinical practice, RBC-mag is commonly used since it measures intracellular magnesium deficiency [57].

### 3.3. Magnesium Supplementation

A double-blind randomized controlled clinical trial has not been done to evaluate the efficacy of magnesium for ADHD. However there is preliminary evidence to suggest that magnesium may be promising to reduce the symptoms of ADHD. There are six reported studies using magnesium as an intervention for ADHD. Of these, three had a control group [58]. The largest study was an observational study of 810 children (5–12 years of age) with symptoms of inattention and behavioral problems (but not with a confirmed diagnosis of ADHD) who were supplemented with 80 mg of magnesium, zinc, eicosapentaenoic acid, docosahexaenoic acid, and gamma-linolenic acid [59]. After 12 weeks of supplementation, there was a reduction in symptoms of inattention as well as hyperactivity/impulsivity. In children with ADHD, one study showed improvement in the clinical symptoms of ADHD with a magnesium-vitamin B6 (Mg-B6) regimen (6 mg/kg/d Mg, 0.6 mg/kg/day vitamin B6), and increased RBC magnesium levels with supplementation. However, there was no statistically significant association between the increase in RBC-mag levels and degree of improvement of clinical symptoms [55].

Although magnesium supplementation normalized RBC-magnesium levels in some studies [55,60], in others, RBC magnesium levels did not normalize after supplementation although hair magnesium levels did improve [61].

Trave showed that children taking stimulants had decreased magnesium intake as compared to healthy controls (222.9 ± 49.8 mg *vs.* 291.2 ± 86.3 mg) [51]. The DRI for magnesium is 250 mg for males and females ages 9–13; therefore, some of these children may be deficient in magnesium.

### 3.4. Safety

Of the trials involving supplementation, only one commented on safety and adverse effects. No serious adverse effects occurred, however, 1.1% discontinued the supplement secondary to intolerance [59]. The most common adverse side effect is diarrhea; at very high dosages magnesium has caused paralytic ileus, metabolic alkalosis, and hypokalemia [62]. Based on animal studies, there is concern that in some individuals, depending on dosage, supplementation may cause increase in aggression [52,53]. It has been suggested that moderate doses (up to 200 mg/day) pass the criteria of being a safe, inexpensive, and possibly efficacious adjunctive treatment for individuals with ADHD [52].

### 3.5. Summary

There is preliminary evidence to suggest that a subgroup of patients presenting with hyperactivity, impulsivity, and inattention may be deficient in magnesium. It is unclear if this is true for only certain regions of the world. Some individuals may be at higher risk for magnesium deficiency (e.g. those with appetite suppression or proton pump inhibitors [63]). In individuals presenting with symptoms of AHD, where there is clinical suspicion for magnesium deficiency, clinicians may consider measuring RBC-mag levels.

## 4. Vitamin D

### 4.1. Basic Functions, Sources, and Relevance for ADHD

In addition to its regulation of calcium and phosphorous in the intestine and stimulation of bone cell mineralization, vitamin D is a neuroactive steroid that has been shown in both animal and human studies to be important for normal brain development [6,64]. Vitamin D receptors and 1α-hydroxylase enzyme (1α-OHase), an enzyme responsible for the formation of the active form of vitamin D, are located throughout the central nervous system. Vitamin D receptors and enzymes are located in neuronal cells of the substantia nigra, hippocampus, hypothalamus, prefrontal cortex, and cingulated gyrus; many of these regions have also been shown to have abnormalities in ADHD [65,66]. There is data to suggest that Vitamin D deficiency during development has deleterious effects on the dopamine system and, in animal models, vitamin D has been shown to be associated with the production of tyrosine hydroxylase, the rate-limiting enzyme for dopamine synthesis [67,68]. Vitamin D may exert its neurological effects through various mechanisms. In animal models, it has been shown that vitamin D is an important factor for the differentiation of developing brain cells, is involved in axonal growth, can increase antioxidants such as glutathione and therefore protect against oxidative stress, and can regulate various neurotrophic factors such as nerve growth factor. Although largely cross-sectional in design, there have been studies demonstrating an association between low vitamin D levels with schizophrenia, depression, and Alzheimer’s Disease [68].

### 4.2. Measuring Vitamin D

Although 1,25-dihydroxyvitamin D is the active form of vitamin D, serum levels are not considered a useful measure of vitamin D status in the body because its half-life is short and it may remain normal even in deficiency secondary to up-regulation of the 1α-OHase enzyme. 25-hydroxyvitamin D (25-OH) is agreed to be the best measure of Vitamin D and reflects both cutaneous synthesis and intake from food and supplements. Although there is debate in the literature regarding optimal levels of Vitamin D, individuals with 25-hydroxyvitamin D levels below 20 ng/mL (50 nmol/L) are considered deficient and levels above 50 ng/mL (125 nmol/L) may cause potential adverse effects. For vitamin D, the current Recommended Dietary Allowance is based on data for bone health. The Institute of Medicine determined in their 2011 report that there was insufficient evidence to show a causal relationship between vitamin D and extraskeletal outcomes. The Recommended Dietary Allowance for individuals ages 1–70 is 600 IU/day. The tolerable upper intake levels for children ages 4–8 is 3,000 IU/day and for children 9 and above is 4,000 IU/day [69].

### 4.3. Sources

Provitamin D molecules in the skin are converted by sunlight to vitamin D. Dietary sources of vitamin D include fatty fish, eggs, butter, liver, and fortified foods like milk [6]. Individuals at increased risk for insufficient amounts of vitamin D include those with darker skin pigmentation, limited sun exposure, kidney disease, liver disease, disorders of malabsorption, and taking some antiepileptic drugs [70].

### 4.4. Evidence for Differential Level

In a case control study done in Qatar, 1,331 children diagnosed with ADHD (ages 5–18) based on clinical diagnosis and rating scales, were matched with children without ADHD. There was a statistically lower level of 25-OH vitamin D (16.6 ± 7.8 ng/mL) in the ADHD group compared with healthy controls (23.5 ± 9.0 ng/mL, *p* < 0.001). These correlations continued to be true after adjusting for BMI and gender (adj. OR = 1.54 with CI of 1.32–1.81, *p* < 0.001). 19.1% of children with ADHD *vs.* 12.7% of healthy controls had 25-OH vitamin D levels of less than 10 ng/mL, indicating severe deficiency. Limitations of the study included that dietary intake was not measured nor amount of time in the sun; therefore, it is unclear if these changes were due to differences in the amount of sunlight exposure, dietary intake, or metabolism of vitamin D. Even in sun-rich countries, vitamin D deficiency exists in part due to limited time outdoors because of extreme climates [71].

In a cross section study, a group in Turkey measured 25-OH vitamin D in children (ages 9 ± 2.2 years old) diagnosed with ADHD (*n* = 60) and compared these to a healthy control group (10.1 ± 3.3 years old). The ADHD group had statistically lower levels of 25-OH vitamin D (20.9 ± 19.4 *vs.* 34.9 ± 15.4 ng/mL). No differences were noted in levels of calcium, phosphorous, or alkaline phosphatase and levels of vitamin D were not different in the three subgroups (inattentive *vs.* hyperactive/impulsive *vs.* combined) [72].

### 4.5. Summary

There are no current studies in children using vitamin D as a treatment for ADHD, but two studies show lower levels of vitamin D in individuals with ADHD. There are vitamin D receptors in key areas of the brain implicated in ADHD, and increasing research showing the importance of this vitamin in brain development. Clinicians may consider measuring 25-OH vitamin D levels in some individuals with symptoms of ADHD, especially in those with other risk factors. 

## 5. Iron

A systemic review of the literature examining the association of iron and ADHD was recently published; the reader is referred to this article for a more comprehensive review [73]. Iron is necessary for the synthesis of catecholames and dopamine. Ferritin is a marker of peripheral iron stores, and can be used to estimate body-iron stores; however, it is not known if it reflects brain iron [73]. Although some studies have shown lower levels of ferritin in children with ADHD, other studies have not found this correlation; therefore, current results are mixed [73,74,75]. There is only one randomized-placebo controlled trial (*n* = 23) evaluating the efficacy of ferrous sulfate (80 mg) for non-anemic children with ADHD and low ferritin levels (<30 ng/mL); supplementation showed some improvement of symptoms of ADHD [76].

Studies have estimated the prevalence of Restless Leg Symptoms (RLS)/RLS symptoms in patients with ADHD to be up to 44% [77]. In 2003, a report from a National Institutes of Health (NIH) consensus conference defined four essential criteria of RLS: urge to move the legs, worse with rest/inactivity, worse in the evening, and relieved by movement. For children, there is an additional criteria of the child expressing leg discomfort in their own words [78]. It is hypothesized that a common dysfunction of the dopamine system accounts for the high comorbidity. There is currently no definitive treatment algorithm for children presenting with RLS [79]. In children with RLS and low ferritin (less than 40 ng/mL), iron supplementation was found to be effective for improving symptoms of ADHD in a small open-label study [80]. Given the high comorbidity, children with symptoms of ADHD should be screened for RLS, and in those with RLS, measuring serum ferritin should be considered.

## 6. Conclusions

Many symptoms of ADHD are addressed with behavioral therapy and medications; however, even with combined treatments, one-third of patients are still symptomatic [4]. Currently there is no evidence to support supplementation as a monotherapy for the treatment of ADHD, however, supplementation may improve medication response and overall well-being, especially in those with deficiencies. Although it is not definitively clear the percentage of children presenting with symptoms of ADHD who have nutrient deficiencies, the existing literature suggests that a subgroup of children with ADHD are at risk for nutrient deficiencies which may play a role in symptomology. In children presenting symptoms of ADHD, clinicians are encouraged to review the dietary history, consider risk factors for zinc, iron, magnesium, and vitamin D deficiency and order RBC-magnesium, 25-OH vitamin D, ferritin, and serum zinc when appropriate (see Table 1).

Key acronyms: 25-OH (25-hydroxyvitamin D), RDA (Recommended Daily Allowance), DRI (Dietary reference intake), UL (Tolerable Upper Intake), EFA (Essential fatty acids), EAR (Estimated average requirement).

**Table 1:** Possible Nutrient Deficiencies in Children with ADHD

Nutrient	Existing Evidence for Differential Levels in Children with ADHD	Children at Additional Risk for Nutrient Deficiency	Laboratory Measurement
Zinc	Yes	vegetarians, poor dietary intake of zinc-rich foods	Serum/plasma zinc and/or hair zinc
Magnesium	Yes	poor dietary intake of magnesium-rich foods (whole grains, nuts, legumes, seafood, green vegetables), consuming medications that decrease magnesium absorption (e.g. proton pump inhibitors)	RBC-magnesium
Vitamin D	Yes	darker skin pigmentation, limited sun exposure, kidney disease, liver disease, disorders of malabsorption	25-OH Vitamin D
Iron	Yes	poor dietary intake of iron	Ferritin
